# Brain Network Modularity During a Sustained Working-Memory Task

**DOI:** 10.3389/fphys.2020.00422

**Published:** 2020-05-08

**Authors:** Marta Moraschi, Daniele Mascali, Silvia Tommasin, Tommaso Gili, Ibrahim Eid Hassan, Michela Fratini, Mauro DiNuzzo, Richard G. Wise, Silvia Mangia, Emiliano Macaluso, Federico Giove

**Affiliations:** ^1^Centro Fermi–Museo Storico della Fisica e Centro di Studi e Ricerche Enrico Fermi, Rome, Italy; ^2^Fondazione Santa Lucia IRCCS, Rome, Italy; ^3^Dipartimento di Neuroscienze Umane, Sapienza Univeristà di Roma, Rome, Italy; ^4^Dipartimento di Fisica, Sapienza Università di Roma, Rome, Italy; ^5^Department of Physics, Helwan University, Cairo, Egypt; ^6^Istituto di Nanotecnologia, Consiglio Nazionale delle Ricerche, Rome, Italy; ^7^Independent Researcher, Rome, Italy; ^8^Institute for Advanced Biomedical Technologies, University of Chieti, Chieti, Italy; ^9^Cardiff University Brain Research Imaging Centre, School of Psychology, Cardiff University, Cardiff, United Kingdom; ^10^Center for Magnetic Resonance Research, Department of Radiology, University of Minnesota, Minneapolis, MN, United States; ^11^ImpAct Team, Lyon Neuroscience Research Center, Université de Lyon, Lyon, France

**Keywords:** functional connectivity, modularity, topology, working memory, connectivity dynamics, brain segregation

## Abstract

Spontaneous oscillations of the blood oxygenation level-dependent (BOLD) signal are spatially synchronized within specific brain networks and are thought to reflect synchronized brain activity. Networks are modulated by the performance of a task, even if the exact features and degree of such modulations are still elusive. The presence of networks showing anticorrelated fluctuations lend initially to suppose that a competitive relationship between the default mode network (DMN) and task positive networks (TPNs) supports the efficiency of brain processing. However, more recent results indicate that cooperative and competitive dynamics between networks coexist during task performance. In this study, we used graph analysis to assess the functional relevance of the topological reorganization of brain networks ensuing the execution of a steady state working-memory (WM) task. Our results indicate that the performance of an auditory WM task is associated with a switching between different topological configurations of several regions of specific networks, including frontoparietal, ventral attention, and dorsal attention areas, suggesting segregation of ventral attention regions in the presence of increased overall integration. However, the correct execution of the task requires integration between components belonging to all the involved networks.

## Introduction

Brain activity is associated, both at rest and during cognitive engagement, with spatial patterns of synchronized, slow (<0.1 Hz) blood oxygenation level-dependent (BOLD) fluctuations which identify different brain networks, namely, neural systems which are coupled together (Rogers et al., 2007). Functional connectivity (FC) can be characterized exploiting various analytical methods and metrics, but it is commonly defined as the temporal correlation between signals from anatomically distinct regions (Friston, 1994; Horwitz, 2003). Consistency of resting-state functional magnetic resonance imaging (fMRI) networks in healthy human adults is well established (Damoiseaux et al., 2006; van den Heuvel and Hulshoff Pol, 2010) and the pathophysiological relevance of network connectivity changes induced by several diseases and aging has been reported (Sala-Llonch et al., 2014; Zhao et al., 2014; Mascali et al., 2015; Brueggen et al., 2016).

However, the current knowledge of modulation properties of networks and function in response to experimental challenges is still incomplete. In particular, little is known about the changes of functional interaction between networks during the execution of a task.

Several studies (Fox et al., 2005; Fransson, 2006; Golland et al., 2008) roughly identified two distinct large-scale systems, based on their observed functional response to stimuli requiring external attention, including working-memory (WM) tasks. One large system is implicated in cognitive functions entailing attentional demand, in particular top-down or goal-directed attention (Corbetta and Shulman, 2002; Fox et al., 2005; Corbetta et al., 2008). This system, usually named task-positive network (TPN) (Fox et al., 2005), includes the dorsal attention network (DAN) and ventral attention network (VAN), the frontoparietal network (FPN), as well as somatosensory and visual areas. The second system, identified as the default mode network (DMN) (Buckner et al., 2008), responds to attention-demanding tasks with a decreased BOLD signal, thus indicating deactivation (Harrison et al., 2008), while being preferentially activated during unconstrained thoughts, introspection, memory retrieval, and self-evaluation of future perspective (Greicius and Menon, 2004; Greicius et al., 2004; Fransson, 2006; Buckner et al., 2008; Fornito et al., 2012).

Spontaneous BOLD fluctuations in TPN and DMN were found to be reciprocally anticorrelated (Fox et al., 2005), and the internal coherency fluctuations were found differently modulated during cognitive functions, often in opposite directions (Lowe et al., 2000; Hampson et al., 2002, 2006; Newton et al., 2007; Gordon et al., 2014; Liang et al., 2016). At first, this competitive nature was thought to foster the switching between internal thought and reaction to external stimuli, to support the efficiency of brain processing (Fox et al., 2005; Fransson, 2006; Golland et al., 2008). Indeed, the degree of anticorrelation between DMN and TPN regions has been associated with faster reaction times during cognitively demanding tasks (Kelly et al., 2008) and better performance in WM n-back tasks (Hampson et al., 2006).

However, other studies supported task-specific cooperation rather than competition between DMN and TPN areas (Newton et al., 2007; Spreng et al., 2010; Bluhm et al., 2011; Leech et al., 2011; Fornito et al., 2012; Bray et al., 2015; Piccoli et al., 2015; Zuo et al., 2018). Cooperative dynamics was found to entail reallocation of flexible areas inside each network, implying a topological reorganization of networks themselves (Bullmore and Sporns, 2009; Braun et al., 2015; Liang et al., 2016). Flexible modules are thought to operate as functionally independent entities in response to specific environmental demands (Newton et al., 2007; Spreng et al., 2010; Leech et al., 2011; Fornito et al., 2012; Bray et al., 2015; Piccoli et al., 2015; Vatansever et al., 2015), and modular reorganization has been shown to be associated with behavior (Leech et al., 2011; Fornito et al., 2012; Braun et al., 2015; Vatansever et al., 2015) and modified by pathology (Crossley et al., 2013; Lerman-Sinkoff and Barch, 2016; Bordier et al., 2018).

In a recent study investigating FC adaptations to a sustained n-back WM task, we have shown that connectivity changes associated with task execution include a widespread modulation of synchronization patterns both within and between brain networks (Tommasin et al., 2018). This widespread change preserved the gross topology of whole-brain connectivity, with the exception of specific areas within some networks, including DMN, FPN, DAN, and VAN. These findings raise the issue of identifying the topological relations between flexible areas that showed adaptation to the task. In the present work, we applied to the same data of our previous study the graph analysis developed by Fornito et al. (2012) to identify the relevance and the behavioral role of the topological reorganization of a set of brain networks during a steady-state working-memory task.

## Materials and Methods

### Subjects

Twenty right-handed Italian-speaking subjects (eight females, age 33 ± 6 years), with no history of neurological or psychiatric disease, participated in the study. All subjects gave written informed consent in accordance with the Declaration of Helsinki and European Union regulations and the Ethics Committee of Santa Lucia Foundation in Rome approved the study. Subjects and data included in this study are the same involved in our previous study (Tommasin et al., 2018).

### Image Acquisition

Data were collected on a head-only 3 T MRI Scanner (Magnetom Allegra, Siemens Healthineers, Erlangen, Germany) equipped with a standard quadrature birdcage coil, used for both transmission and detection. Functional images were acquired via a gradient-echo planar imaging (GE-EPI) sequence (TR = 2100 ms, TE = 30 ms, FA = 70°, voxel size 3 × 3 × 2.5 mm^3^) lasting 24 min and 38 s for a total of 704 volumes (four dummy scans included). The slices were positioned starting from the vertex and covered the whole cerebrum. High resolution T1-weighted images were acquired for anatomic reference and tissue segmentation purpose using a Magnetization Prepared Rapid Acquisition Gradient Echo (MPRAGE, TE = 4.38 ms, TR = 2000 ms, FA = 8°, voxel size 1.33 × 1.33 × 1 mm^3^).

### Stimulation Paradigm and Task Performance

The experimental setup and stimulation paradigm are fully described in Tommasin et al. (2018). Briefly, BOLD data were acquired within a block-design paradigm, composed of alternated epochs of open-eyes resting state and sustained auditory WM task (4 min and 54 s each, starting with a resting-state epoch). The auditory WM task involved continuous n-back trials at “high” load (two-back) or “low” load (one-back). Each trial was composed of a 500-ms window, in which subjects were aurally presented with a pseudorandom vowel, and a subsequent 1600-ms response window, during which subjects had to report whether the current vowel was the same as the one presented one stimulus prior (one-back) or two stimuli prior (two-back). Subjects responded via an MRI compatible two-button keyboard, with one button reserved for positive responses (matching trial) and one button reserved for negative responses (not matching trial). During the entire functional run, subjects were asked to maintain the gaze on the center, marked by a 1° circle over a uniform black background.

Each subject underwent two functional runs during the same experimental session, with epoch ordering: rest/one-back/rest/two-back/rest or rest/two-back/rest/one-back/rest.

Subjects responses to the WM task were monitored at runtime and recorded for subsequent correlation analyses with modularity metrics (see below). For each subject, performance during each task epoch was evaluated in terms of accuracy computed as the percentage of valid responses on the number of trials (response was considered as valid, if it was both correct and given during the 1600-ms response window).

### Image Preprocessing and Processing

Functional and structural MRI data were pre-processed using FC toolbox (CONN 17.c) (Whitfield-Gabrieli and Nieto-Castanon, 2012) and analyzed with dedicated in-house routines based on Matlab R2013a (The Mathworks Inc., Natick, MA, United States) and AFNI (Cox, 1996). Detailed descriptions of preprocessing steps are reported in Tommasin et al. (2018). Briefly, functional images underwent motion and slice-timing correction, and were normalized to the Montreal Neurological Institute (MNI) space (voxel size 2 × 2 × 2 mm^3^). Spurious variance was further removed by regressing out motion-derived parameters and aCompCor signals (Behzadi et al., 2007), by applying a bandpass filter (0.008–0.09 Hz) and by censoring motion-contaminated volumes (Jo et al., 2013). Finally, spatial smoothing was applied with an 8 × 8 × 8 mm^3^ FWHM Gaussian kernel. An unsmoothed version of the data was retained for FC computation.

Each functional run was split in five epochs. The first resting-state epoch was discarded from analyses and was used only for cortical parcelation. Specifically, using the smoothed data of the first epochs (resting epochs), the cortex was parcelated in 350 regions of interest (ROIs) by means of group level ROIs based on the similarity among voxel time courses (Craddock et al., 2012). ROIs were then classified into seven large-scale networks using the atlas from Yeo et al. (2011). We retained ROIs belonging to four networks of interest (205 ROIs) which included the DAN, DMN, the FPN, and the VAN networks. These four networks showed the most conspicuous traces of reorganization in spite of the overall preserved topology that we had reported on the same dataset (Tommasin et al., 2018), and are thought to respond during the execution of a WM task, as described in Section “Introduction.”

The remaining four epochs, i.e., one-back, two-back, and two resting states, were used to extract epoch-related FC. For each ROI and epoch, an average time-course was extracted from unsmoothed data and correlated to each other ROI time-course, leading to an ROI-to-ROI (205 × 205 sized) correlation matrix. Fisher’s Z transformation was applied to the correlation matrix to improve normality. Then, correlation matrices computed from homologous epochs, as well as from task epochs with different load levels, were averaged for each subject in order to obtain two single resting-state and task-related matrices for each subject. These connectivity matrices were fed into the graph analysis procedure described below. The common treatment of both task levels conforms to choices adopted in our previous study, justified by the observation that the network behavior was indistinguishable between the levels; in particular we did not observe reproducible FC effects of task load (for details, see Tommasin et al., 2018).

### Graph Analysis

We used the modularity analysis proposed by Fornito et al. (2012) to characterize networks reconfiguration during rest and task conditions. Modules are defined as clusters of nodes showing greater FC within the clusters than with the rest of the brain.

For each subject and condition (i.e., rest and task), we modeled interactions between networks as a graph of 205 nodes, representing regions of parcelation constituting each network. We identified the optimal modular decomposition, maximizing a quality function (Q) (Rubinov and Sporns, 2011) reflecting the goodness of partition, using the Louvain method (Blondel et al., 2008), implemented in Brain connectivity Toolbox^1^, with resolution parameter set to unity. We defined the *optimal modular decomposition* as the partition with the maximum Q over 10,000 iterations of the algorithm.

The modularity, as expressed by Q, gives an index of degree of modular segregation in the graph (Lebedev et al., 2018). Q is close to one when there are few edges between modules and high density inside modules, while Q is close to zero when the number of connections between modules is comparable to those of a random graph. We compared Q between conditions by means of a two-sample paired *t*-test.

For each subject and each node in the graph, from the optimal modular decomposition, we computed the within-module strength, *z*, and the diversity coefficient, *h*, for rest (named *z*_*R*_ and *h*_*R*_) and task (named *z*_*T*_ and *h*_*T*_) condition, respectively. The within-module strength quantifies the node’s intramodular connectivity and it was calculated as the z-score-transformed degree of centrality within the module. Formally, for each node *i*, *z*_*i*_ is defined as

$$z_{i}=\frac{s_{i}\,\left(m_{i}\right)-\bar{s}\,\left(m_{i}\right)}{\sigma_{\bar{s}\,\left(m_{i}\right)}}$$

where *m*_*i*_ is the module containing node *i*, *s*_*i*_(*m*_*i*_) is the within-module node strength (i.e., the sum of the within-module weights of node *i*), and $\bar{s}\,\left(m_{i}\right)$ and $\sigma_{\bar{s}\left(m_{i}\right)}$ are the mean and standard deviation of the within-module strength of all nodes in module *m*_*i*_, respectively (Fornito et al., 2012). The diversity coefficient describes the node’s distribution of intermodular connectivity and it was calculated with the normalized Shannon entropy, specifically

$$h_{i}=\frac{1}{\log m}\,\sum_{u\,M}p_{i}\,\left(u\right)\,\log p_{i}\,\left(u\right)$$

where $p_{i}\,\left(u\right)=\frac{s_{i}\,\left(u\right)}{s_{i}}$, *s*_*i*_(*u*) is the strength of node *i* in module *u*, and *m* is the number of modules in the partition *M* (Shannon, 1948; Rubinov and Sporns, 2011; Fornito et al., 2012). Thus, nodes with high *z* values are suggested to represent local intramodular hubs, while nodes with high *h* values, presenting an even distribution of connectivity across modules, are suggested to support functional integration between modules (Fornito et al., 2012).

We further characterized modularity at group level using the following procedure (Fornito et al., 2012). For each subject and condition, from the optimal modular decomposition, we constructed a 205 × 205 co-classification matrix (cC) in which the element cC_*ij*_ = 1 if nodes *i* and *j* belong to the same module and cC_*ij*_ = 0 otherwise. For each condition, we derived a group consistency matrix as the summation over all subjects of the co-classification matrices. Thus, the group consistency matrix represents the node co-classification frequency across subjects. The group consistency matrix was then subjected to a further modular decomposition in order to obtain an optimal group modularity matrix, for rest and task condition, respectively (also in this case the algorithm was iterated 10,000 times for testing degeneracies in the data and the resolution parameter was set to its default value). From the two optimal group modularity matrices, we determined the nodes that changed module membership from rest to task condition and we mapped them onto brain volume.

Similar to the subject-level analysis, for both conditions, we characterized the role of each node in the network at the group level by means of *z* and *h* computed on the optimal group modularity (Guimera and Amaral, 2005). At group level, nodes with high *z* values have highly conserved modular membership across subjects, while nodes with high *h* values have a variable membership identity across participants (Fornito et al., 2012).

### Statistical Inference

To investigate the behavioral role of the degree of integration between modules, the diversity coefficient *h* of each node during task was correlated with subjects’ accuracy in task execution via Pearson’s correlation coefficient. Results were corrected for multiple comparisons across nodes via false discovery rate (FDR; Benjamini and Hochberg, 1995).

In order to assess the statistical significance of the topological configuration changes between rest and task conditions, we used a permutation test, using an approach similar to what proposed by Alexander-Bloch et al. (2012) and applied by Bordier et al. (2018). Specifically, we used the normalized mutual information (NMI, Kuncheva and Hadjitodorov, 2004) to assess the similarity between rest and task optimal group modularity matrices and compared it to a null distribution. If the change in topological configuration was driven by the experimental condition, then the experimental data should have yield a lower NMI value than those generated from the null distribution. To build the null distribution we repeated 10,000 times the process to generate the optimal group modularity matrices, each time randomly relabeling the rest and task conditions (permutations constrained via within-subject exchangeability blocks). The *p*-value was calculated as the number of times in which the NMI was lower than the NMI of the experimental data, divided by the number of permutations.

Unless otherwise stated, numerical results are given as mean ± standard deviation.

## Results

### Behavioral Data

Behavioral data from three subjects could not be recorded for technical problems, leaving a total of 17 subjects for the behavioral analyses. For each subject, performances were evaluated in terms of accuracy, computed as the percentage of valid responses on the number of trials. Performances did not show significant change between the first and the second run of each session (paired *t*-test, *p* > 0.57) and was higher for the lower WM load, as expected (97% one-back, 84% two-back; *p* < 10^–5^, paired *t*-test). For complete details, see Tommasin et al. (2018).

### Modularity

The permutation test revealed a significant change in the topological configuration between rest and task (*p* = 0.0017). The optimal modular decomposition resulted in modularity across subjects of *Q* = 0.36 ± 0.06 at rest and *Q* = 0.31 ± 0.09 at task. The degree of segregation was significantly different across conditions (paired *t*-test, *N* = 20, *t* = 3.9, *p* = 0.001).

**FIGURE 1:**
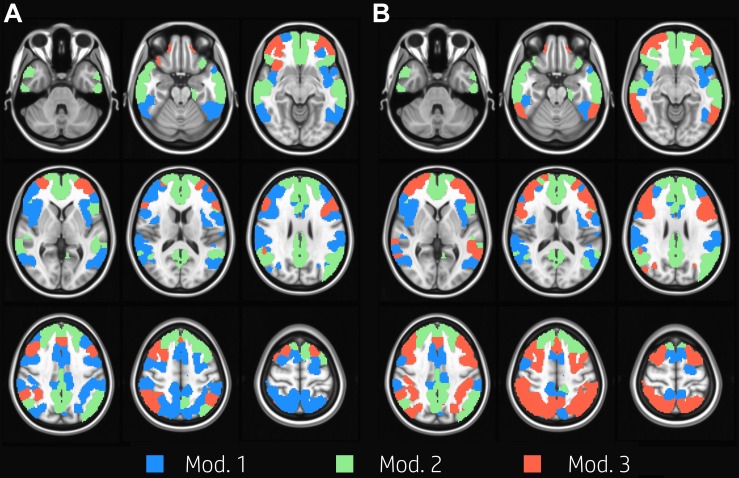
Group modularity. Evenly spaced axial slices covering the whole brain for group modular membership at rest **(A)** and task **(B)**. Nodes are color-coded according to the module membership. Three group-level modules were identified for both task and at rest condition; module 2 was clearly preserved between task and rest, and it is mainly composed of nodes in the DMN, while the other two modules showed large reorganizations between rest and task.

We identified three group-level modules both at task and at rest (Figure 1). The overall network structure showed a reorganization of nodes’ module membership during the execution of the task, resulting in a Jaccard index of 0.71 between the two functional conditions. While module 2, which was mainly composed of nodes of the DMN (Table 1), was clearly preserved between task and rest, the other two modules showed large reorganizations between rest and task. In particular, the resting condition was characterized by the presence of a large module (module 1, 96 nodes) mainly composed of VAN, DAN, and FPN nodes, and of a substantially smaller module (module 3, 27 nodes), whose nodes laid mainly in FPN. At task, the size of the two modules was more uniform (52 and 76 nodes, respectively). Module 1 was mainly composed of VAN nodes and to a lesser extent of DAN nodes. Module 3 grouped almost all FPN nodes and the majority of DAN nodes. In other words, FPN was equally shared between modules 1 and 3 at rest, and was mainly included in module 3 during the execution of the WM task, while DAN was mainly included in module 1 at rest and was shared between module 1 and 3 at task.

**TABLE 1:** Number of nodes and network membership of each module in both functional conditions.

Condition	Module	Number of nodes	Network membership			
			
			DAN	DMN	FPN	VAN
Rest	**1**	96	36	4	21	35
	**2**	82	1	77	2	2
	**3**	27	1	6	20	0
Task	**1**	52	11	6	2	33
	**2**	77	2	69	3	3
	**3**	76	25	12	38	1

**FIGURE 2:**
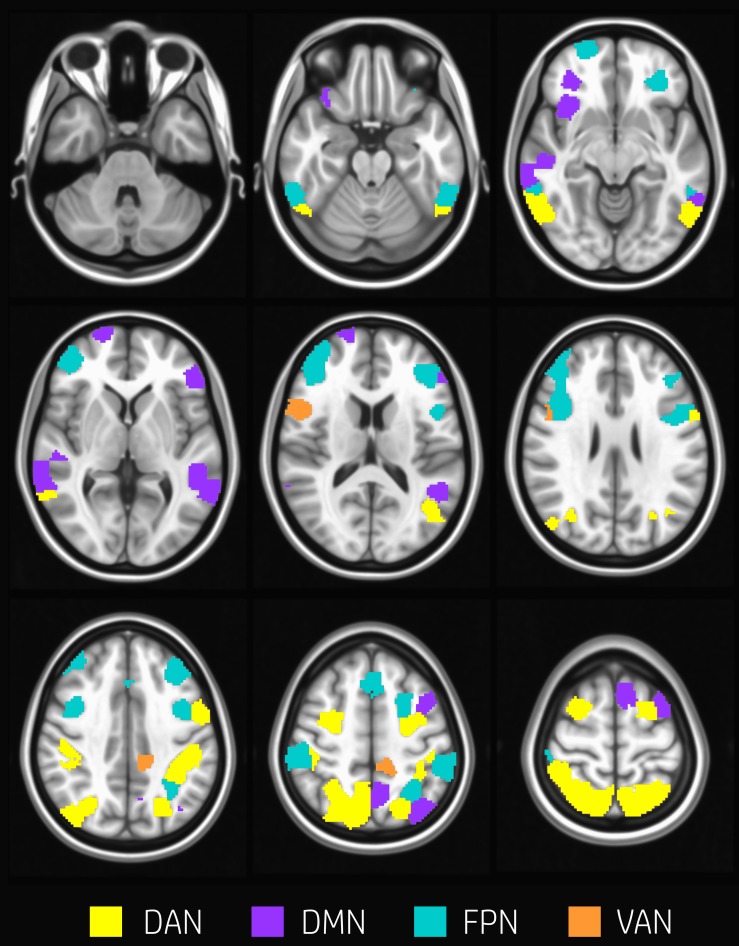
Nodes changing modularity. Evenly spaced axial slices covering the whole brain for nodes changing modular membership between rest and task conditions. Nodes are color-coded according to network membership. About 29% of nodes (60 nodes) changed membership between rest and task. Nodes changing their membership were mainly located in occipital cortex, temporal gyrus, postcentral and precentral gyrus, frontal gyrus, frontal pole, and cingulate gyrus.

About 29% of nodes (60 nodes) changed modular membership between rest and task. These nodes represent a substantial amount of flexibility in modular composition. Nodes changing their membership were mainly located in the occipital cortex, temporal gyrus, postcentral and precentral gyrus, frontal gyrus, frontal pole, and cingulate gyrus (Figure 2).

### Nodes Functional Role

**FIGURE 3:**
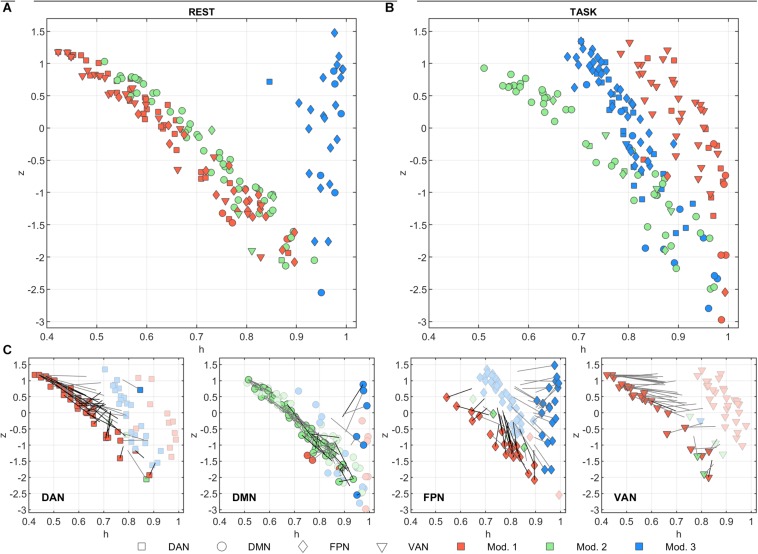
Node functional role in the *h*/*z* plane. The within-module strength (*z*) is plotted as a function of the diversity coefficient (*h*) for each of the 205 nodes, separately for **(A)** rest and **(B)** task conditions. Nodes are color-coded according to module membership and shape-coded according to network membership. The change in module memberships and in the *h*/*z* positions can be appreciated in **(C)** where the *h*/*z* plots are shown separately for each network and separately for rest (darker color) and task (lighter color) conditions. Gray lines starting from rest nodes describe the trajectory vector of the node between task and rest. Note, however, that the length of the vector is halved for clarity purposes. Thus, each node position at task can be identified by doubling the length of the trajectory. Nodes that change module memberships between rest and task conditions are marked with darker trajectories.

Figures 3A,B summarize the functional role of the nodes at rest and task in the *h/z* plane (diversity coefficient and within-module strength, respectively). The trajectories of each node between task and rest are shown in Figure 3C.

At rest, *z* showed a strong inverse correlation to *h* in modules 1 and 2 (Figure 3A, *r* = –0.98, *p* < 10^–10^ for both). In module 3, the correlation was not significant but tended to be positive (*r* = 0.18, *p* = 0.37). Module 3 was indeed characterized by consistently high values of *h*. These nodes mainly belong to FPN and were located in the occipital and frontal cortex. At task, *z* and *h* were still inversely correlated both in module 1 and 2 (respectively *r* = –0.59 and *r* = –0.95, *p* < 10^–10^ for both). During task, *z* and *h* of nodes belonging to module 3 become inversely correlated as well (*r* = –0.89, *p* < 10^–10^). The correlation changed significantly from rest to task for modules 1 and 3 (z = –10.8, *p* < 10^–10^ and z = 5.6, *p* < 10^–5^, respectively, test on Fisher’s Z transformed correlations). In other words, functional role of nodes in module 2 did not change between conditions, while module 1 and module 3 moved toward each other in plane *h/z*, going both toward higher *z* values, while *h* on average increased in module 1 (from 0.62 ± 0.15 to 0.905 ± 0.066) and decreased in module 3 (from 0.958 ± 0.032 to 0.797 ± 0.070). Taking into account the network composition of each module (see above), this behavior was determined by DAN, FPN, and VAN nodes in module 1, and by FPN and (to lesser extent) DMN in module 3 (see Figure 3, bottom plots).

### Behavioral Scores

**FIGURE 4:**
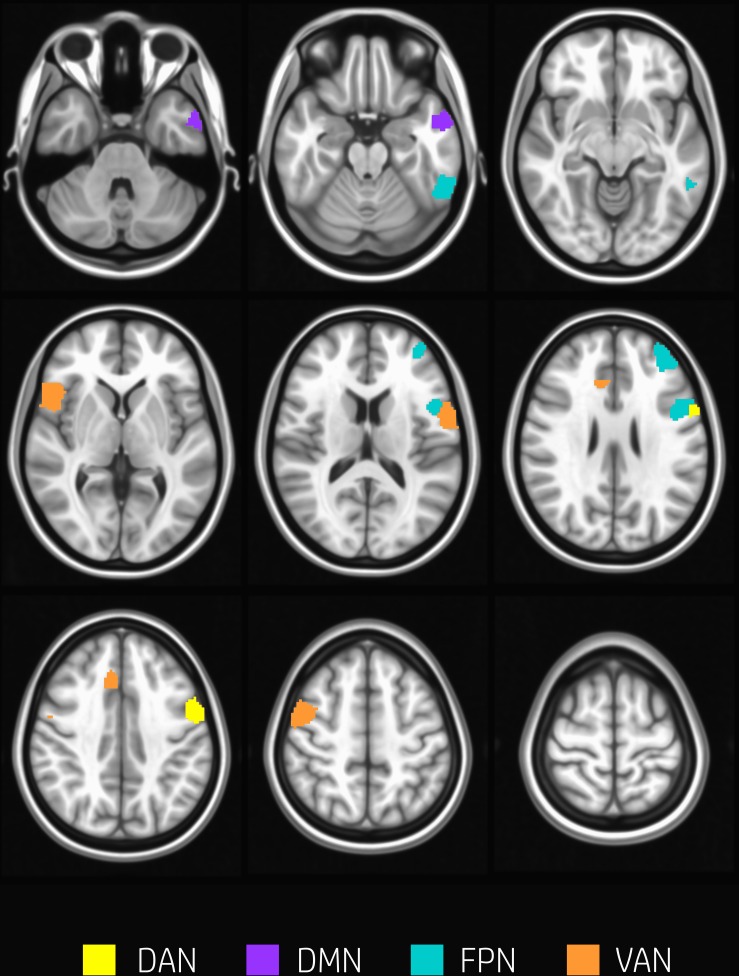
Nodes correlating with performance. Nodes showing a significant positive correlation between *h*_*t*_ and subjects accuracy (*p* < 0.05, FDR corrected) are found in temporal and frontal areas including regions of the left inferior and middle temporal gyrus, and regions across the precentral sulcus, the anterior part of the precentral gyrus, and the posterior part of the middle and inferior frontal gyrus (bilaterally). A more rostral section of the left middle frontal gyrus and a section of the anterior cingulate gyrus were involved as well. Nodes are color-coded according to network membership.

The overall degree of brain segregation, as quantified by Q, correlated inversely with subject accuracy, both at rest and task (*r* = –0.61 and *r* = –0.54, respectively. *p* < 0.05 for both). The change of segregation between rest and task, while significant in itself (see above), was not correlated with the behavioral performances. Significant positive correlations were found between *h*_*t*_ and subject accuracy (*p* < 0.05, FDR corrected) in temporal and frontal areas. These are summarized in Table 2 and shown in Figure 4. These areas included regions of the left inferior and middle temporal gyrus, and regions across the precentral sulcus, including the anterior part of the precentral gyrus, and the posterior part of the middle and inferior frontal gyrus (bilaterally). A more rostral section of the left middle frontal gyrus and a section of the anterior cingulate gyrus were involved as well.

**TABLE 2:** Nodes showing significant correlation between *h*_*T*_ and subject accuracy (*p* < 0.05, FDR corrected).

Node	Network	Module		Label	*h*_*T*_	
				
		Rest	Task		*r*	*p*
36	DAN	1	3	L Precentral Gyrus	0.7573	0.0004
188	VAN	1	1	L Precentral Gyrus (inf fro gy)	0.8199	0.0001
137	FPN	1	3	L Precentral Gyrus	0.7041	0.001
79	DMN	2	2	L Middle Temporal Gyrus	0.7020	0.001
130	FPN	1	3	L Inferior Temporal Gyrus	0.6902	0.002
161	FPN	1	1	L Middle Frontal Gyrus	0.7716	0.0003

*For each node, the table reports network, module membership at rest and task, anatomical label, and *r* and *p* of correlation.*

## Discussion

In our previous study, based on the same dataset, we found that the continuous performance of a sustained WM task is associated with large and widespread FC changes (Tommasin et al., 2018). The overall brain topology did not change between rest and task, but we found some evidence of task-driven dissociation between regions within VAN and modest network reorganization involving nodes within FPN, DAN, and DMN as well. In the present study, we investigated the exact features of these topological changes and the corresponding functional and behavioral correlates.

During rest, DAN, VAN, and part of FPN coexisted in a common module, suggesting intrinsic dynamic collaboration of these networks (Damoiseaux et al., 2006; Fox et al., 2006; He et al., 2009). Modularity analysis showed that cognitive engagement entails a dynamic reorganization of several brain areas belonging to the investigated networks. The degree of brain segregation was significantly different between rest and task conditions, and task-performance was associated with a significant increase of brain global integration. Notably, the Q parameter, related to network segregation, was inversely correlated to subjects’ accuracy in both conditions. This result is consistent with previous studies, using different techniques (Kitzbichler et al., 2011; Wen et al., 2015; Liang et al., 2016; Shine et al., 2016; Hearne et al., 2017; Zuo et al., 2018), and confirms that a brain topology suitable for integration of information is a prerequisite for the correct performance of a WM task.

Several previous works reported that task-driven (Braun et al., 2015; Hearne et al., 2017; Zuo et al., 2018) or dynamic (Shine et al., 2016; Fransson et al., 2018) reconfiguration of brain modular structure during task is driven by the flexibility of areas within FPN, DMN, DAN, and VAN. In our modular analysis, we found a high level of reorganization for nodes belonging to the TPNs, in particular for regions in frontal and temporal cortices (Figure 2). The only module overall unaffected by the task-performance was module 2, that included most of the DMN.

Module 1 included virtually the whole VAN (at least 89% of nodes), both at rest and at task, while participation of DAN and FPN nodes to this module changed between conditions. Specifically, at rest module 1 included all DAN and half of FPN nodes, at task it lost its FPN coverage and around 2/3 of the DAN nodes. On the other hand, FPN nodes converged to a single module, suggesting that the task is associated with a consolidation of FPN function.

This interpretation is corroborated by the analysis of changes in the *h* and *z* parameters. Indeed, nodes in DAN, VAN, and DMN changed their functional role rather uniformly, in agreement with the overall behavior of the pertaining modules. However, the split modularity of FPN at rest was mirrored by opposite changes in *h*, with nodes of modules 1 and 3 converging to a common module characterized by intermediate *h* values (Figure 3, bottom). These changes, taken together, indicate a task-dependent segregation of the functions carried out by the DAN and FPN nodes versus activities relying on VAN nodes. Considering the overall increase of integration during task, this effect indicates a tighter integration between areas belonging to DAN and FPN networks.

Our results show a clear task-driven dissociation between DAN and VAN: almost all the nodes within these networks were in the same module at rest, while during task 2/3 of DAN nodes transitioned to a third module that also included the FPN nodes. Indeed, albeit both structural (Umarova et al., 2009) and rsfMRI (Fox et al., 2006; He et al., 2007) studies have shown that DAN and VAN were plainly discernible, it has been repeatedly suggested that the attention control requires flexible and continuous collaboration between these networks (Fox et al., 2006; He et al., 2007; Macaluso, 2010; Chica et al., 2011; Vossel et al., 2014). The dissociation that we observed here could reflect the switching between reflexive (bottom up) and voluntary (top-down) attention during the task. Accordingly, many studies suggested that DAN and VAN have opposite pattern of activity during the execution of a WM task (Corbetta et al., 2000; Shulman et al., 2009; Asplund et al., 2010).

While the main topological reorganization associated with task-execution concerned the segregation of VAN modules from FPN and 2/3 of the DAN nodes, the inter-modular integration at task was found to be crucial for accurate performance. It is worth noting that eight of nine of the nodes where *h*_*T*_ correlated with behavior belong to either module 1 or 3, including three nodes that changed module during task (from 1 to 3).

This result confirms that the performance during WM was related to the capability of preserving intramodular integration in spite of modular reorganization.

The fact that no node had z (i.e., within-network hub properties) correlated to performance may indicate that integration between-modules is more crucial than within-network connections in the current WM task. From a network perspective, the execution of the WM task requires integration between DAN and FPN, but the performance was boosted by further integration with VAN.

Indeed, one of the key components of WM processing is the capacity of actively maintaining information no longer available and to manipulate this information for usage over short delays (Moser et al., 2018). This requires the integration of neuronal circuits on large scale, including regions in dorsolateral prefrontal cortex, parietal cortex, and dorsal anterior cingulate cortex (Liang et al., 2016). All of these regions are represented in the nodes characterized by correlation between *h*_*T*_ and performances (Table 2 and Figure 4).

## Conclusion

In conclusion, we have shown that the execution of an auditory WM task is associated with a switching between different topological configurations of FPN, VAN, and DAN nodes, involving a segregation of VAN nodes in the presence of increased overall integration. The correct execution of the task requires integration between components belonging to all the involved networks.

## Data Availability Statement

The datasets of this article are not publicly available because the authors lack the ethical consent to do that. Requests to access the datasets should be directed to the corresponding author.

## Ethics Statement

The protocol of the study was approved by the Ethics Committee of Santa Lucia Foundation. All subjects gave written informed consent in accordance with the Declaration of Helsinki and with the European Union regulations.

## Author Contributions

MM acquired and processed the data prepared the figures and wrote the manuscript. DM acquired and processed the data and prepared the figures. ST acquired and processed the data. TG designed the study and programmed the stimulation. IH and MF performed the experiment. MD, RW, SM, and EM discussed the results and the manuscript and helped in the interpretation of the results. FG designed the study, interpreted the results, and coordinated the research. All authors edited the text and approved the final version.

## Conflict of Interest

The authors declare that the research was conducted in the absence of any commercial or financial relationships that could be construed as a potential conflict of interest.

## Footnotes

1 https://sites.google.com/a/brain-connectivity-toolbox.net/bct/
